# Cervical spinal injury associated with patient positioning during percutaneous nephrolithotomy

**DOI:** 10.1016/j.eucr.2026.103425

**Published:** 2026-03-31

**Authors:** Rithik Kumar, Theresa Olmstead, Manoj Monga

**Affiliations:** a Department of Urology, University of California San Diego, United States; b University of California San Diego, 9500 Gilman Dr, 92093, La Jolla, CA, United States

## Abstract

Percutaneous nephrolithotomy (PCNL) is classically performed with patients in the prone position. We describe a 70-year-old male with a history osteoarthritis of C-spine who underwent a left percutaneous nephrolithotomy for a 2.2 cm staghorn calculus and subsequently suffered an iatrogenic cervical spine injury requiring surgical intervention. This case suggests screening imaging in patients with cervical osteoarthritis or selection of a supine approach in these patients should be considered.

## 1 Introduction

While patient positioning during PCNL for kidney stones has not shown to impact success rates,^1^ most urologists use prone positioning.^2^ In determining between prone and supine positioning, stone complexity,^3^ patient anatomy and comorbidities,^4^ surgical workflow requirements, and surgeon experience are considered. However, there is no published literature that provides a formal ranking or framework for determining PCNL positioning in complex patients with competing considerations. This case report describes an iatrogenic cervical spine injury sustained during a proned PCNL that required surgical intervention, suggesting screening imaging in patients with cervical osteoarthritis or selection of a supine approach in these patients should be considered.

## 2 Case presentation

A 70-year-old male with history of cerebrovascular accident (on 81mg aspirin), osteoarthritis of C-spine, obstructive sleep apnea (on CPAP) prostate cancer (s/p RALP) was seen for bilateral nephrolithiasis and booked for a staged left percutaneous nephrolithotomy (for 2.2cm staghorn) and right extracorporeal shock wave lithotripsy (11mm interpolar calculus). Prior to intubation for the PCNL, anesthesia performed their routine H&P and noted the patient's pre-existing cervical spine rigidity. Though it was not deemed severe enough to preclude standard general anesthesia and proned positioning, the anesthesiologist for the case did bring attention to this during positioning and intubation. In an abundance of precaution, a GlideScope was used for intubation as to avoid having to extend the patients' neck. The surgical team worked closely and judiciously with anesthesia during the positioning of this patient given the neck rigidity noted preoperatively, making several alterations to ensure the most neutral position of the neck. Specifically, a Dupaco Adjustable ProneView Protective Helmet System with mirror served as the primary structural supportfor the patient's head and neck. This face-contoured device was used due to its ability to stabilize the head in a non-flexed, non-extended and its demonstrated superiority in minimizing surface pressure on the patient's face by 29% compared to other non-countered Prone Positioner^5^. The procedure itself lasted 1.5 hours and was otherwise uneventful.

**Fig. 1:**
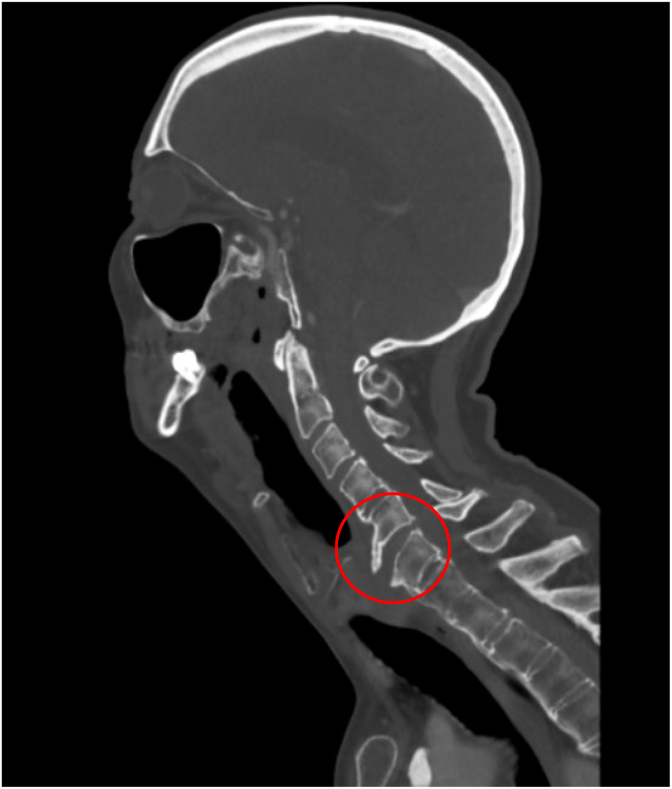
Sagittal stroke CT angiogram head and neck demonstrating separation of a C5-6 osteophyte (red circle) previously stabilizing C-spine.

**Fig. 2:**
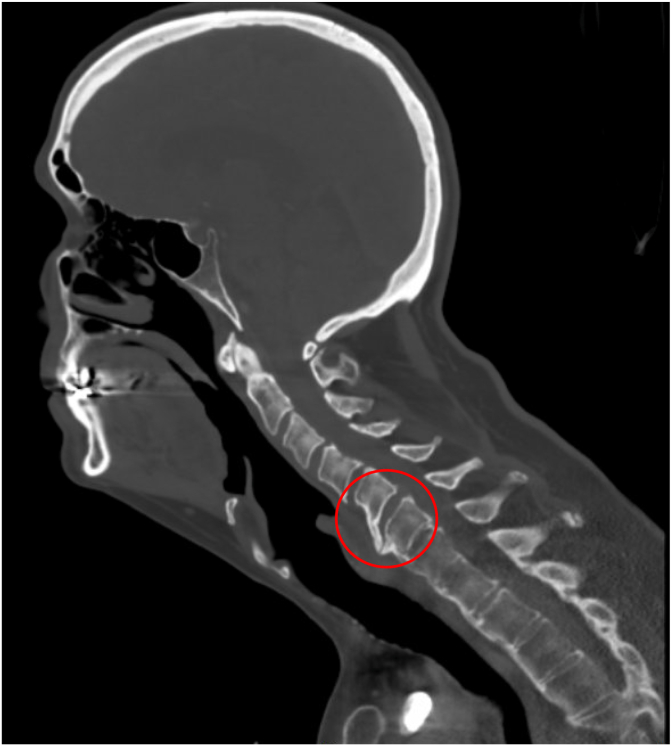
Sagittal stroke CT angiogram head and neck demonstrating a C5-6 osteophyte (red circle).

**Fig. 3:**
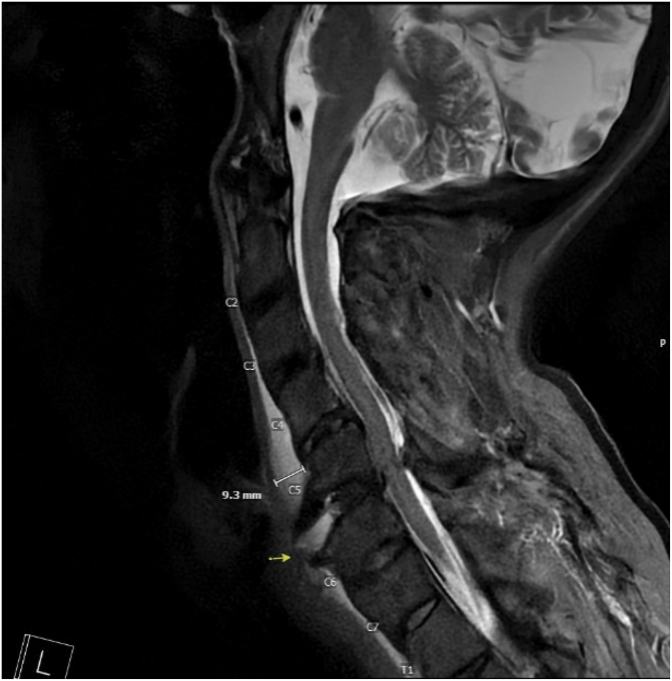
MRI Cervical spine w/o contrast demonstrating C5-6 traumatic grade 1 retrolisthesis with ligamentum flavum infolding/disruption.

Patient was brought to PACU in stable condition. Upon waking up, the patient's wife noted a significant right side facial droop. Stroke code was called immediately. Neurology followed the patient to the CT scanner and performed a thorough evaluation that ultimately excluded acute stroke and confirmed a diagnosis consistent with Bell's Palsy. On CTA, however, there was an incidental finding of widening of C5-6 (Fig. 1) along with a distracted anterior osteophyte (Fig. 2) that did not clinically correlate to the neurologic deficit that prompted the stroke code initially. Neurosurgery was consulted and recommended C-collar and MRI which then revealed an acute fracture of an osteophyte that was previously stabilizing the patient's C-spine and traumatic grade 1 retrolisthesis at C5-6 (Fig. 3). Due to the unstable nature of this injury, neurosurgery suggested and proceeded with anterior cervical discectomy and fusion (ASDF) via anterior cervical spine fusion.

Consistent follow-up with patient 7-months post-ASDF along with review of CT scans, revealed bones are healing ahead of anticipated schedule with bony bridging across the surgical site. The patient reported posterior muscular fatigue which was consistent with his healing stage that required activation and recruitment of musculature. Follow-up was scheduled in three months with x-rays to monitor progress and a CT scan was tentatively scheduled at 1-year post-complication.

## 3 Discussion

This is the first reported case of cervical spine injury as a complication of PCNL, with most positioning-related injuries involving peripheral nerve injuries or musculoskeletal issues rather than c-spine trauma.^6^ While current medical literature lacks case series or evidence documenting cervical spine injury post-PCNL, improper head and neck support, excessive flexion or extension, and inadequate padding could propagate cervical spine stress.^7^

In this case, the patient presented with iatrogenic cervical injury, found incidentally upon workup of post-op, left sided facial droop. CTA imaging ultimately revealed incidental widening of C5-6 likely due to an acute fracture of a critical osteophyte. Although such injuries have not been extrapolated upon in the literature, the theoretical risk is suggested to come from positioning mechanics. Moreover, patients with cervical spine instability may be at higher risk during positioning and as such pre-op attention is necessary to ensure safety.

Known risk factors to positioning include stone complexity, patient anatomy and comorbidities, and surgical workflow requirements, all of which may contribute to early identification and mitigation of injury. Furthermore, management strategies depend on structured screening of patient risk factors, interdisciplinary team coordination, and repositioning or integration of assisted devices.

This case highlights an unmet need in establishing clear, risk-stratified guidelines on patient positioning and patient stabilization strategies, that can reduce long-term complications. Perhaps, a brief in-clinic evaluation by the urologist should be performed as part of the surgical planning processes. Although, pre-op discussions occurred and intra-operative safety measures were implemented, the patient still endured a potentially catastrophic injury that could have left him permanently paralyzed. Advancements towards standardizing positioning recommendations or contraindications in patients with multiple comorbidities could help guide positioning based on highest risk adverse events.

## 4 Conclusion

Cervical spine injury is an uncommon yet critical complication of PCNL that can result in significant morbidity if unrecognized and not promptly addressed. This case underscores the value of evidence based, risk-stratified patient positioning guidelines and safe intervention in patients with multiple comorbidities. Prompt identification and appropriate mitigation are crucial to maximizing patient safety.

## CRediT authorship contribution statement

**Rithik Kumar:** Writing – review & editing, Writing – original draft. **Theresa Olmstead:** Writing – review & editing. **Manoj Monga:** Writing – review & editing.
